# Recent Developments of Functional Scaffolds for Craniomaxillofacial Bone Tissue Engineering Applications

**DOI:** 10.1155/2013/863157

**Published:** 2013-09-15

**Authors:** Yukihiko Kinoshita, Hatsuhiko Maeda

**Affiliations:** Department of Oral Pathology, School of Dentistry, Aichi Gakuin University, 1-100 Kusumoto-cho, Chikusa-ku, Nagoya 464-8650, Japan

## Abstract

Autogenous bone grafting remains a gold standard for the reconstruction critical-sized bone defects in the craniomaxillofacial region. Nevertheless, this graft procedure has several disadvantages such as restricted availability, donor-site morbidity, and limitations in regard to fully restoring the complicated three-dimensional structures in the craniomaxillofacial bone. The ultimate goal of craniomaxillofacial bone reconstruction is the regeneration of the physiological bone that simultaneously fulfills both morphological and functional restorations. Developments of tissue engineering in the last two decades have brought such a goal closer to reality. In bone tissue engineering, the scaffolds are fundamental, elemental and mesenchymal stem cells/osteoprogenitor cells and bioactive factors. A variety of scaffolds have been developed and used as spacemakers, biodegradable bone substitutes for transplanting to the new bone, matrices of drug delivery system, or supporting structures enhancing adhesion, proliferation, and matrix production of seeded cells according to the circumstances of the bone defects. However, scaffolds to be clinically completely satisfied have not been developed yet. Development of more functional scaffolds is required to be applied widely to cranio-maxillofacial bone defects. This paper reviews recent trends of scaffolds for crania-maxillofacial bone tissue engineering, including our studies.

## 1. Introduction

Critical-sized bone defects in the craniomaxillofacial region due to tumor excisions, injuries, congenital disorders, and advanced resorptions of the alveolar bone after teeth loss can cause damage to their structures, leading to noticeable deformity and dysfunction. They are generally reconstructed with autogenous bone graft, allogeneic bone graft, xenograft, or alloplastic materials [1–5]. However, each has certain advantages and disadvantages and poses limitations in simultaneously fulfilling both morphological and functional restorations of the defects. In particular, for reconstruction of critical-sized bone defects, it is generally agreed that application of free vascularized bone graft from distant sites including fibula, iliac crest, scapula, and radius is the most reliable procedure [1, 2, 4]. Nevertheless, this graft procedure has several disadvantages such as restricted availability, donor-site morbidity, prolonged hospitalization, and rehabilitation. There are also limitations in regard to fully restoring the complicated three-dimensional structures in the craniomaxillofacial bone, especially in the jawbone using dentures or dental implants. Conversely, bone substitutes made of inorganic materials such as metals are available with little morbidity, but the associated foreign body response throughout the patient's lifetime makes dentures and dental implants impossible.

The ultimate goal of the craniomaxillofacial bone reconstruction is the regeneration of the physiological bone that simultaneously fulfills both morphological and functional restorations. It is a healthcare problem worldwide now. Developments of tissue engineering in the last two decades have brought such a goal closer to reality [6–8].

The basic strategy of bone tissue engineering is to corporate into the target site one or more of the fundamental elements necessary for bone formation such as scaffolds, osteoprogenitor cells (mesenchymal stem cells), bioactive factors, or genes to stimulate cellular proliferation and differentiation, while guiding the tissue-repairing function of the living body. A tissue engineering approach to the craniomaxillofacial bones provides several potential benefits including the lack of donor-site morbidity, no limitation of availability, no risk of immunoreactivity, and disease transmission. 

There are two primary strategies in bone tissue engineering. The first is to implant an acellular biodegradable scaffold with/without a bioactive factor into the target site, leading to recruitment of local mesenchymal stem cells and/or osteoprogenitor cells that would regenerate the bone. This strategy is called the *in situ* tissue engineering where the scaffold should play the role of a spacemaker or an osteoconductive matrix for the ingrowth of cells from the surrounding tissues. Therefore, the scaffold might be applied to relative small defects. The second is to implant a scaffold with mesenchymal stem cells (MSCs) and/or osteoprogenitor cells of an external source into the bone-defected site. Furthermore, the latter strategy has two approaches. The first approach is to directly transplant MSCs and/or osteoprogenitor cells combined with a scaffold (external scaffold) into the bone-defected site, which is a kind of an *in situ* tissue engineering. Autogenous particulate cancellous bone and marrow (PCBM) are used as the source of osteoprogenitor cells and MSCs. In this approach, the scaffold plays the role of a framework [9]. The second approach is to transplant MSCs that are isolated (usually from the patient), expanded *ex vivo*, seeded on adequate three-dimensional scaffolds (internal scaffolds), and proliferated in controlled culture conditions (extracorporeal tissue engineering or tissue engineering in a narrow sense) [10]. Such a scaffold acts as a carrier of the cells and temporary matrix while the cells produce the extracellular matrix (ECM) that is required for bone formation. This approach is attractive for bone regeneration in aged patients or large bone defects because it requires only a small amount of bone marrow or an other tissue. For any strategy or approach, the scaffold is a key for achieving successful bone regeneration. In addition, it should be considered that the craniomaxillofacial region has a unique anatomic location with complicated environments and special functional requirements. For instance, there are complicated three-dimensional structures in this region, there is a contaminated environment—like the oral cavity—and, in particular, strong mechanical strength is required for the jawbone to perform a proper function such as chewing. Therefore, the scaffold should be selected corresponding to each strategy or purpose. This paper describes new trends including our studies, while highlighting the use of scaffolds for craniomaxillofacial tissue engineering.

## 2. Scaffolds for Acellular Bone Tissue Engineering

### 2.1. Guided Bone Regeneration Membrane (GBR Membrane)

Unfavorable conditions of the alveolar ridge such as a thin or low-volume alveolar bone, due to tooth loss often preclude placement of dental implants and the insertion of dentures, even when damage of bone defects is not so large. Therefore, reconstruction of the alveolar ridge is an important topic in the field of implants and prosthetic dentistry. Guided bone regeneration (GBR) has attracted attention as a promising method [11–15].

The principle of the treatment is the placement of a barrier membrane between the bone-defected site and the gingival tissue. The membrane plays an essential role as a spacemaker in preventing the rapid influx of soft tissue cells and guides new bone formation into a desired shape, while osteoprogenitor cells and osteoblasts are recruited from the surrounding bone. Requirements of the GBR membrane include biocompatibility, maneuverability, flexibility, and vascularization and have enough mechanical strength to withstand gingival compressive forces or occlusion forces during bone regeneration. So far, no resorbable membranes composed of synthetic polymer or metal, and resorbable (degradable) membranes made of collagen or synthetic polymers have been developed; several of them have been already commercially used as membranes for GBR [16–18]. However, no ideal membrane has yet been developed. The ePTFE or the TR-PTFE (ePTFE reinforced with titanium) had been commonly used because of convenience. Despite reports of the high predictability, there are some disadvantages. The main disadvantage of this membrane is a high rate (~43%) of exposure, which can cause bacterial contamination and early removal of the membrane, associated with diminished results [19–21]. Also, even if there are no complications, a second surgery is needed in order to remove the membrane after the target defect is healed, bringing physical and economical burdens to the patient. Nowadays, manufacture has been stopped on account of the manufacturer. These drawbacks are driving researchers to develop a bioabsorbable membrane. Two materials are mainly used to manufacture the bioabsorbable membrane: collagen and synthetic polymers. Collagen has many advantages over other materials, including allowing early wound stabilization, being cell adhesive, and having sufficient permeability to permit nutrient transfer [22]. However, collagen membranes begin to degrade by the enzymatic activity of macrophages, polymorph nuclear leukocytes, and bacteria at locations after membrane placement, and, thus, they lose the ability to resist collapse [23, 24]. The mechanical strength is too poor to provide space for bone formation. Although several cross-linking techniques have been proposed to retard the degradation of native collagen membranes [25, 26], bone grafts have to be used together to prevent collapse of collagen membranes. Also, it was reported that GBR using demineralized bone matrix (DBM) and collagen membranes caused favorable bone formation [27]. Furthermore, GBR using DBM-calcium sulfate without the membranes was reported [28]. However, the number of comparisons was limited. Therefore, further investigation is required to determine whether the favorable osteogenic effects originated from the DBM. Conversely, bioresorbable synthetic polymers such as poly(L-lactic acid) (PLLA), poly(glycolic acid) (PGA), polycaprolactone (PCL), trimethylene carbonate (TMC), and their copolymers have been used as membrane materials. Among these, the membrane made of PGA, poly(LA-co-GA) (PLGA), or PGA/TMC is used commercially. Although they do not require a second surgery, they present limitations regarding their ability to provide space for bone formation, early/late absorption, mechanical strength, and inflammatory reaction during biodegradation [16–18, 29]. To overcome the limitations of biodegradable synthetic polymers while maintaining their advantages, polymer-calcium phosphate composites have been investigated [30–32]. Inorganic materials, such as calcium phosphates, are expected to provide rigidity to the soft polymer, the osteoconductivity, the PH buffering effect in the surrounding tissue, and the X-ray impermeability making it possible to monitor the GBR membrane after implantation.

**Figure 1:**
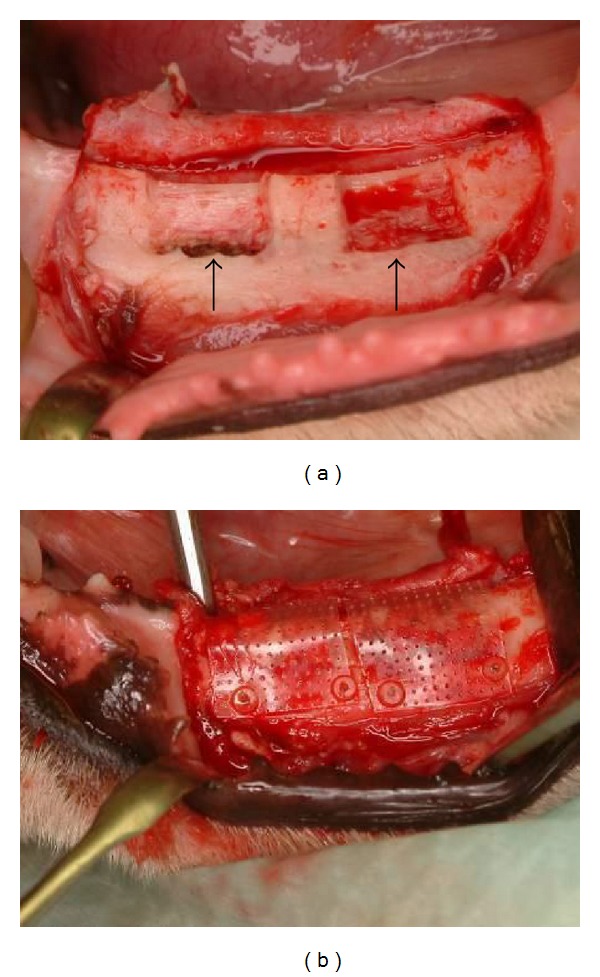
Guided bone regeneration with PLGC macroporous membrane in lateral bone defects of a canine mandible [33]. Clinical appearance of the surgically created bone defects and membrane placement. (a) Intraoperative view of the two lateral bone defects created in the mandible, and (b) view of the PLGC macroporous membrane closely adapted to the bone and stabilized with PLLA pins.

**Figure 2:**
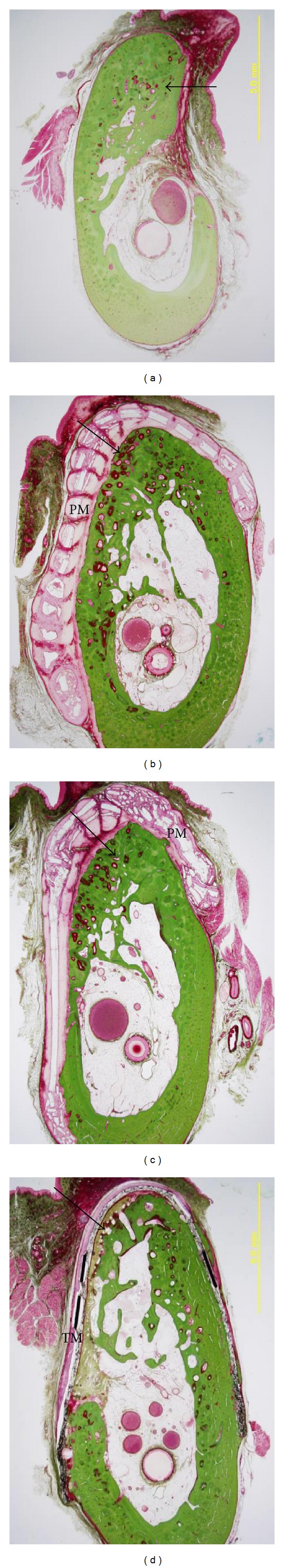
Histological microphotographs of coronal sections at 6 months postoperatively [33]. The Villanueva-Goldner staining: (a) control (virgin) group, (b) GBR using PLGC membrane, (c) GBR using PLGC+bone chips group, and (d) GBR using TR-PTFE membrane. PM: PLGC macroporous membrane. TM: TR-PTFE membrane, and arrow: regenerated bone.

Kinoshita et al. manufactured a novel macroporous biodegradable GBR membrane made of poly(L-lactide-co-*ε*-caprolactone) (75 : 25 mol%) containing 30% beta-tricalcium phosphate (*β*-TCP) [P(LLA-CL)/*β*-TCP] and evaluated the effects of the P(LLA-CL)/*β*-TCP membrane on bone regeneration in a full-thickness saddle-type defect of the mandibular alveolar bone ridge of dogs [32]. The results revealed that a macroporous P(LLA-CL)/*β*-TCP membrane had enough mechanical strength to endure soft tissue compressive forces and sufficient maneuverability to form a desired shape by thermoplasticity at 70°C, provided a desirable space for bone formation, and had limited inflammatory reaction in the surrounding tissue. However, most of the membrane will remain 6 months postoperatively. In many clinical situations, a resorption period not exceeding beyond 6–12 months is mandatory in order not to lose the advantages of resorbability [10]. Further research is needed to decrease the biodegradation period of synthetic polymer membranes with the remaining mechanical characteristics. Then, authors et al. fabricated poly(lactic acid-coglycolic acid-co-*ε*-caprolactons) (75 : 1 : 24 mol%) (PLGC) macroporous membrane (pores: 0.4 mm in diameter; 1.2 mm spacing center to center; thickness: 0.3 mm). L-lactic acid, glycolic acid, and *ε*-caprolactone provide improvement of rigidity, biodegradability, and toughness for the membrane, respectively [33]. Then, we evaluated its effect as a GBR membrane in the alveolar ridge of canines and compared it with a TR-PTFE membrane (Figure 1). The results revealed that there was more bone augmentation at all experimental sites than at control sites not using the membrane in defects. Although the volumes of new bones at the defect sites covered with the PLGC membrane were less than those at the defect sites covered with TR-PTFE membrane, bone density of the regenerated bone was significantly higher at sites covered with the PLGC membrane than at sites covered with the TR-PTFE membrane. There was no difference in volume and density of the regenerated bone between the PLGC macroporous membrane group with autogenous bone chips and those without ones. Histological analysis verified the presence of well-vascularized loose connective tissues in the pores of the PLGC membrane and the fragmentation and resorption of the membrane 6 months postoperatively (Figure 2). The macroporous bioresorbable PLGC membrane effectively facilitates bone regeneration with no bone grafts in GBR procedures.

### 2.2. Bone Replacing Substitutes

For the purpose of replacing with natural bones, a variety of bone substitutes consisting of inorganic and organic materials have been developed as alternatives to autogenous bone grafts [34–41]. The properties of the ideal bone substitute include biocompatibility, availability, easy moldability, compressive strength as the base material for new bone formation, biodegradability, resorbability, replacement by normal bone, and osteoconductivity. So far, the bone substitute that clinically satisfies these conditions has not been developed.

#### 2.2.1. Calcium Phosphate Ceramics

Among bone substitutes, it is widely agreed that calcium phosphate-based porous ceramics are excellent components for bone substitutes because they have similar properties to those of bone, and they also improve the bioactive features by different modifications.

The primary constitutions (60%) of the natural bone are calcium phosphate minerals. The minerals are present as apatite crystals, primarily hydroxyapatite. It is generally agreed that the osteoconductive property of the bone substitute is provided by calcium phosphate (CaP) ceramics. They are available in a variety of products due to differing chemical compositions (Ca: P) and forms [35, 41, 42]. So far, dozens of calcium phosphate materials including hydroxyapatite (HAp), or *β*-tricalcium phosphate (TCP), multiphasic bio-glass, biphasic calcium phosphate (BCP), and octacalcium phosphate (OCP) have been developed and both investigated *in vitro* and *in vivo* [43–56]. These materials demonstrate to enhance migration and adhesion of osteoblasts *in vitro*, and they promote bone tissue formation, bonding to bone *in vivo*. Based on their evidence, some of them are applied in clinical cases.

Porous sintered hydroxyapatite blocks or particles have been used within bone-defected sites for a long time. However, hydroxyapatite (HAp) has a very high elastic modulus far different from that of bone tissue, and it tends to be brittle, not easily molded. Additionally, it is hardly resorbed, or the resorption is very slow, which increases the risk of infection and exposure in oral and maxillofacial regions [37]. Nowadays, individual use of HAp decreases in the oral and maxillofacial regions. Conversely, *β*-TCP has been developed as bioresorbable CaP, and the porous blocks and granules are applied in the clinical cases [34, 37, 44, 45, 47, 48]. Its compressive and tensile strength is nearly equivalent to that of the cancellous bone [41]. Although *β*-TCP is replaced by bone *in vivo*, less new bone is laid down than *β*-TCP is resorbed [41]. It may take more than one year for *β*-TCP to replace the natural bone. Also, *β*-TCP bone substitutes are usually provided in the form of blocks or granules as well as Hap; thus, they are not easily moldable. These causes still limit clinical applicability.

BCP is a mixture of *β*-TCP and HAp of varying amounts [37, 46, 50, 57]. As BCP is resorbed *in vivo*, it releases calcium and phosphate ions into the microenvironment of the implanted site; these ions can be used for new bone formation [50]. Szpalski et al. fabricated BCP bone substitute using a custom-designed 3D microprinting process [37]. When these BCP scaffolds were implanted into a critical-sized alveolar defect in rats either empty or seeded with rhBMP-2, they demonstrated the capability of inducing new bone formation [58].

Recently, octacalcium phosphate is attractive as a promising candidate for resorbable bone substitute [48]. Octacalcium phosphate is advocated to be a biological precursor of hydroxyapatite [59] in bone and tooth. The synthetic granular OCP is converted to the apatitic phase *in vivo* [60] and has been shown to promote differentiation and maturation of osteoblasts [61, 62]. In addition, OCP increases attachment of osteoblasts to the scaffold compared with HAp and *β*-TCP [52], and the implanted OCP granules are resorbed and replaced by a newly formed bone to greater extent than those of HAp and *β*-TCP [48]. However, its brittleness makes it difficult to maintain its shape without restraint. Kamakura et al. developed a composite sponge constructed of OCP and porcine atelocollagen (OCP/collagen) and showed that OCP/collagen sponge significantly enhanced bone regeneration more than the implantation of OCP alone, *β*-TCP/collagen composite, or HAp/collagen composite, when implanted into a critical-sized calvarial defect rat model [51]. Moreover, the efficacy of bone regeneration by OCP/collagen sponge was confirmed in various canine bone defect models [53–56]. The results of the clinical trials are expected.

#### 2.2.2. Synthetic Polymer-Ceramic Composites

Aliphatic polyesters such as PGA, PLA, PCL, and their copolymers are most commonly researched, and several polyesters have been widely used as biodegradable pins and screws as well as surgical suture materials for many years. However, these polymers pose poor osteoconductive capacity and less compressive modulus compared with native bone tissues. Therefore, they have a limitation for use as biodegradable bone substitutes. Conversely, although calcium phosphate-based ceramics offer excellent osteoconductivity, they fail mechanically due to brittleness and are not maneuverable. Then, to overcome the disadvantages of each material while maintaining the advantages, a variety of polymer-ceramic composites have been fabricated and investigated both *in vitro* and *in vivo* [63–70]. The most commonly researched ceramics in polymer-ceramic composites for bone substitutes are Hap and *β*-TCP particles due to their biomimetic and osteoconductive properties, and high-modulus dispersed micro, or nanoscale constituents have been shown to improve the mechanical strength of polymer scaffolds [64–67].

Zhang et al. repaired the critical-sized defects with porous nanohydroxyapatite/polyamide composite blocks in an experimental study in rabbit mandibles [67]. The defects were completely occupied by new bone with density comparable with that of the host bone at 24 weeks. Significant difference was found between nHA/PA groups and blank controls regarding the X-ray opacity over the whole period. The porous nHA/PA composite promoted bone formation of the defect, particularly in the early stage. Davies et al. developed a biodegradable composite scaffold with a pore size and interconnecting macroporosity similar to those of the human trabecular bone [69]. The scaffold is fabricated by a process of particle leaching and phase inversion from PLGA and two calcium phosphate (CaP) phases of which the first is a particulate within the polymer structure and the second is a thin ubiquitous coating. The osteoconductive surface CaP abrogates the putative foreign body giant cell response to the underlying polymer, while the internal CaP phase provides dimensional stability in an otherwise highly compliant structure. Due to the highly interconnected macroporosity and an ability to wick up blood, the scaffold acts as both a clot-retention device and as an osteoconductive support for the host bone growth. They employed this scaffold in human patients to maintain alveolar bone height following tooth extraction and to augment alveolar bone height through standard sinus lift approaches, and they showed that the scaffold served to regenerate sufficient bone tissue in the wound site to provide a sound foundation for dental implant placement [69]. Furthermore, the individualized PLGA/TCP composite scaffold is fabricated, based on alveolar bone defects using a computer-aided low-temperature deposition manufacturing system [70]. This three-dimensional-shaped scaffold was identical to the patient-specific alveolar bone defects. The scaffold biocompatibility was confirmed by attachment and proliferation of the human bone marrow mesenchymal stem cells, and the mechanical properties were similar to those of the adult cancellous bone.

As seen above, bone substitutes are usually provided in the form of blocks or granules, and there are limitations to a clinical application in the craniomaxillofacial region where restoration of the complex form is required.

In the early 1980s, researchers discovered self-setting calcium phosphate cements (CPCs), which are bioactive and biodegradable grafting materials in the form of powders and liquids. Both phases after mixing form viscous paste that after being implanted sets and hardens within the living body as mainly either a hydroxyapatite or a blushite [71]. Hardened CPC is structurally similar to the mineral component of bone and is supposed to become slowly resorbed and simultaneously transformed into a new bone [72–76]. In general, a powder of CPC is formed by a combination of one or more calcium orthophosphates. The cement setting reaction is a dissolution and precipitation process, and the entanglement of the precipitated crystals is the mechanism responsible for cement hardening. CPC has been used to treat compression fracture of the hip, vertebrae, distal radius fractures [77–79], or crania-maxillofacial bone [72, 80, 81]. The chief advantage of CPC is that its form is an easily shaped paste that can be directly injected or pressed into a bone defect during a surgical procedure. It is rigid enough to retain its shape and position, and it can be contoured so that it replaces the lost bone and restores its original shape. Bone augmentation appears to be a very promising application field for CPC.

Although CPC has excellent biocompatibility and osteoconductivity, it is resorbed too slowly *in vivo* [82]. It is desirable that most of the implanted CPC is resorbed and replaced by normal bone after 6 months. Leaving CPC in the healing site too long compromises the clinical outcome of an oral-implant placement because CPC is fragile and is likely to fracture under loading. The slow resorption rate of the current varieties of CPC is due to its dense structure that is not porous enough to allow bone-marrow cells and other cells that generate a new bone to penetrate into the interior of the material [83]. The convenient CPC is intrinsically nanoporous and does not contain an interconnected network of micropores [84]. Therefore, it allows the transport of nutrients and fluid, but it is too small to allow osteoclasts to enter and resorb the CPC.

CPC is broken down and eliminated from an implant site by two mechanisms. One is an active resorption, mediated by the cellular activity of macrophages, osteoclasts, and other types of cells [85–87]. The other is a passive chemical process in which the CPC is either dissolved [88] or broken down by chemical hydrolysis [89]. Therefore, the rate of resorption depends on the porosity of CPC [89]. Bioresorption of the conventional CPCs that are lacking macropores must take place layer-by-layer on the surface, from outside to inside. This substantially slows the resorption process in CPC [90]. One way to increase the resorption rate is to introduce a network of interconnected macropores into the CPC [91, 92]. Microspheres are made of three components: poly(lactic-coglycolic acid) (PLGA), gelatin, and poly(trimethylene carbonate) (PTMC), and they were focused on the introduction of macroporosity in CPC [93–97]. PLGA microspheres inside the CPC, are broken down slowly, preventing cells from rapidly invading the CPC and thus delaying internal bone formation. Therefore, enzymatically degrading polymers (e.g., gelatin and PTMC) were preferred to be introduced into the CPC. Especially natural polymers like gelatin would be the preferred material because it does not express antigenicity in physiological conditions and is completely resorbable [93]. Kasuya et al. fabricated the CPC (Biopex-R) gelatin powder composite containing macropores with interconnectivity and showed that this composite increased the new bone area at the implanted sites in bone defects of distal femurs of rabbits compared with CPC only [98]. We also fabricated an injectable, macroporous calcium phosphate/gelatin cement by mixing CPC and gelatin microparticles (CPC/gelatin), and we implanted the composite paste in the saddle-type bone defects created in the canine mandible for 6 months (unpublished). Micro-CT and histological analysis verified that new bone formation was observed in the internal and peripheral area of the residual implants in CPC/gelatin groups, in contrast with the CPC-alone group in which new bone formation was observed around the periphery of residual implants and no further ingrowth into the implant was observed. CPC was almost resorbed in the 90 wt% CPC to 10 wt% gelatin microparticles group, and it was completely resorbed and replaced by a new bone in the 85 wt% CPC to 15 wt% gelatin microparticles group. New bone replacement was significantly better in the sites treated with CPC containing gelatin microparticles than in those treated with CPC alone. Initial compressive strength of the 90 wt% CPC to 10 wt% gelatin microparticles is compared with that of the cancellous bone. These results indicate that the CPC/gelatin bone cement may be a promising bone substitute for craniomaxillofacial bone defects (unpublished). However, CPC resorption characteristics should be further investigated carefully before clinical use. Also, incorporation of growth factor in to CPC/gelatin should be explored in order to promote bone formation [92–102].

Li et al. showed that the controlled release of rhBMP-2 could be improved in a composite bone substitute with rhBMP-2-loaded gelatin and CPC, and healing of osteoporotic bone could be increased by factors released from a CPC [100]. The most promising direction of the future developments of CPC formulations is obviously seen in their functionalization by incorporation or impregnation of various growth factors.

### 2.3. Scaffolds for Drug (Growth Factors) Delivery System

It is known that various bioactive factors (signals) are involved in natural bone healing [103]. Bone tissue engineering using growth factors is very attractive due to having no patient morbidity. Local administration of growth factors to promote bone formation has been investigated in several preclinical and clinical models [32, 104–109].

Although bone formation is an extended process beyond several weeks, the half-life of the growth factor is short in the physiological environment. The efficacy of the bioactive factor depends on whether its adequate dose can be provided over the appropriate therapeutic time frame at the site of bone formation. For this performance, scaffold or carrier that traps the bioactive molecule and sustains release at the site of bone formation plays a significant role as well as the bioactive molecule.

Regarding the growth factors that enhance bone formation, the bone morphological proteins (BMPs), the basic fibroblast growth factors (bFGFs), the platelet-derived growth factors (PDGFs), and the insulin-like growth factors (IGFs) are identified and isolated [35, 104, 110, 111]. Among them, BMPs have been investigated for bone forming ability by many researchers since Urist discovered their ectopic osteoinductivity in 1965 [112]. Recombinant human (rh) BMP-2, -4, and -7 have been shown to stimulate the osteoprogenitor differentiation into mature osteoblasts and to repair critical-sized defects in experimental studies [113–117]. Furthermore, these osteoconductive effects have also been investigated in clinical studies, showing their definite osteoconductive potentials [118–122]. However, at present, the indications approved by the FDA for rhBMP-2 and BMP-7 are nonhealing tibial fractures, spinal fusion procedures in the orthopedics region, and only sinus augmentation in the craniomaxillofacial region [120, 122].

As the carrier of BMPs, the absorbable collagen sponge made of type 1 collagen (ACS) is currently used and approved by the FDA. However, several drawbacks still remain to be resolved. The therapeutic regimens using ACS release rhBMP-2 at an initially high rate after which the rate declines rapidly [103]. Therefore, an excellent high dose of BMPs is needed to induce bone formation. When endogenous BMPs are naturally released, mere nanogram quantities of the proteins per gram of bone matrix are enough to trigger the bone repair cascade. However, microgram quantities of BMPs per matrix material are needed to gain a bone-inductive effect [123]. At clinical cases, several milligram quantities of recombinant human BMPs are used with ACSe. It is not only cost prohibitive, but it is also a cause of side effects such as severe inflammation, soft tissue swelling, excessive amount of ectopic bone formation, and increased bone resorption [124–126]. These side effects are especially problematic in craniomaxillofacial bone reconstruction in which complex bone architecture is required and maintenance of adjacent soft tissue function as well as a favorable cosmetic outcome is needed. In the living body, ECMs locally bind, store, and release endogenous growth factors at an adequate amount and at the right timing.

There is to a limitation to control release of growth factors using a collagen sponge. There is a pressing need to develop a drug delivery system (DDS) that can contain a dose of a growth factor sufficient to promote bone healing over several weeks and release it slowly and continuously into the bone-defected site with well-controlled kinetics mimicking the ability of natural ECMs. In order to achieve this performance, researchers have incorporated variable growth factors into natural polymers, synthetic polymers, or ceramics by physical entrapment or chemical immobilization [35, 103].

#### 2.3.1. Natural Polymers

Natural polymers, except for collagen hydrogel, alginate hydrogel, and gelatin hydrogel, showed promising results in preclinical and clinical studies [32, 127–130]. For example, alginate hydrogels provided sustained release of BMPs, VEGFs, or other proteins over several days to weeks by locally binding to the alginate matrix, and they, promoted moreover, the segmental bone repair [129]. Furthermore, Kolambkar et al. developed spatiotemporal control of the regenerative process [130]. They utilized a hybrid protein delivery system that comprised of two parts: a perforated cylindrical polycaprolactone nanofibrous mesh that spatially confines the defect region and a functionalized alginate hydrogel that provides temporal BMP growth factor release kinetics. BMP-mediated functional restoration of critical femoral defects in a rat was compared with the current clinical standard of collagen delivery. The hybrid delivery system significantly increased bone regeneration and improved biomechanical function compared with the collagen sponge delivery. The nanofiber mesh tube was essential to promote maximal mineralized matrix synthesis, prevent extra-anatomical mineralization, and guide an integrated pattern of bone formation. 

Conversely, Tabata and Ikada developed the gelatin hydrogel DDS that sustained release of growth factor incorporated into gelatin hydrogels, resulting in effectively exerting the biological functions of the growth factor [127]. Gelatin, a denatured collagen, is obtained by acid and alkaline processing of collagen isolated from bovine bone. This processing affects the electrical nature of collagen, yielding gelatin with different isoelectric points (IEPs). When mixed with positively or negatively charged gelatin, an oppositely charged protein ionically interacts to form a polyion complex. The biodegradable hydrogel matrices are enzymatically degraded in the living body with time. The degradation is controllable by changing the extent of cross-linking, which, in turn, produces hydrogels with a different water content. The time course of protein release is in good accordance with the rate of hydrogel degradation. The protein incorporated into gelatin hydrogel is released as a result of its biodegradation. This gelatin hydrogel DDS releases the protein drug under the maintenance of biological activity.

Yamamoto et al. fabricated hydrogels of gelatin with different water contents that were prepared through glutaraldehyde cross-linking of gelatin with an isoelectric point of 9.0 under varied reaction conditions, and they implanted gelatin hydrogels incorporating BMP-2 into the segmental critical-sized bone defect of rabbits [128]. The gelatin hydrogels incorporating BMP-2 exhibited significantly high osteoinduction activity compared with that of the free BMP-2, although the activity depended on the water content of hydrogels. In addition, significantly higher bone mineral density enhancement was observed for the gelatin hydrogel with a water content of 97.8 wt% than that with the lower or higher water content. These results show that the biodegradable gelatin hydrogel is a promising controlled release carrier of BMP-2 for bone regeneration. However, gelatin is mechanically weak as a scaffold for bone tissue engineering. Combination with calcium phosphate may produce a structurally stronger scaffolding material.

*β*-TCP has been investigated as a carrier for BMP-2 release [131]. Takahashi et al. showed that a sponge composed of gelatin and *β*-TCP suppressed the deformity of the gelatin sponge and enhanced proliferation and differentiation of MSC [132]. BMP-2 adsorbed on the surface of *β*-TCP can be released through the detachment accompanied with the pore surface erosion, since *β*-TCP is biodegradable. We can say with fair certainty that BMP-2 is released from the composite material consisting of gelatin hydrogel and *β*-TCP based on the *in vivo* degradation of both gelatin and *β*-TCP.

Authors et al. fabricated a sponge biomaterial consisting of a biodegradable mixture of gelatin (IEP: 9.0) and a 50 wt% of *β*-TCP (gelatin/*β*-TCP sponge), and they implanted the gelatin/*β*-TCP sponge that bound bone morphogenetic protein-2 (BMP-2) in critical-sized mandibular bone defects in rats [133]. There is significantly higher osteoinductive activity at bone-defect healing sites treated with gelatin-*β*-TCP sponges incorporating BMP-2 than at the sites treated with sponges not incorporating BMP-2. Histologically, a gelatin-*β*-TCP sponge incorporating BMP-2 was replaced entirely with a new bone that contains bone marrow and that is connected to the original bone. The results show that gelatin/*β*-TCP incorporating BMP-2 is osteogenic enough to promote bone. In addition, the gelatin/*β*-TCP composite provides a mechanically stronger and more maneuverable property to the scaffold during surgical procedures.

**Figure 3:**
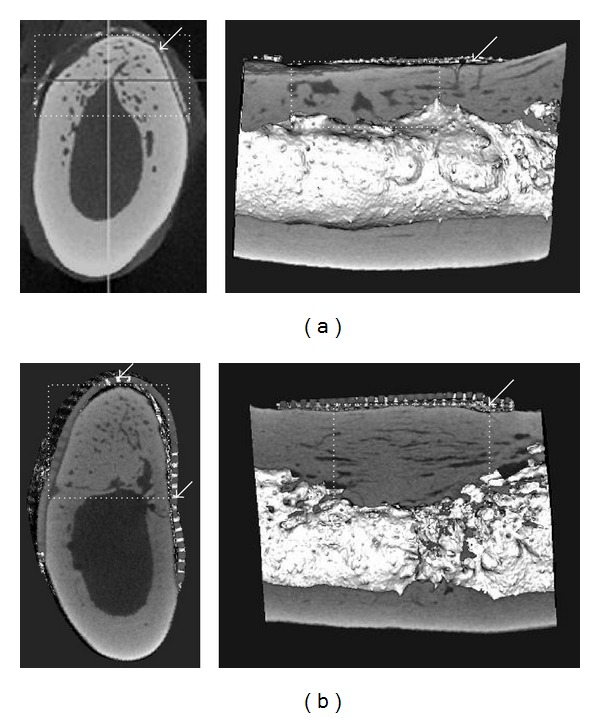
Alveolar bone regeneration using poly(L-lactide-co-*ε*-caprolactone)/*β*-TCP membrane and bFGF-gelatin sponge in the mandible of a canine [32]. Micro-CT images of frontal and sagittal sections in the mandible 6 months postoperatively. (a) Group using only GBR membrane, and (b) group using membrane and bFGF-gelatin sponge circles: regenerated bone; and arrow: GBR membrane.

**Figure 4:**
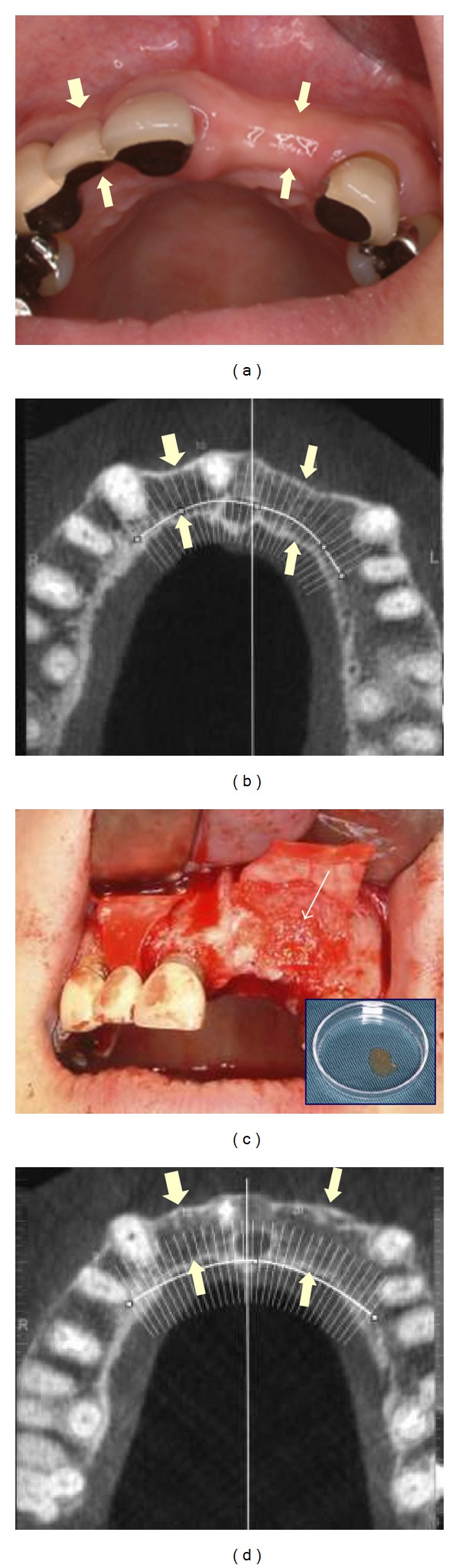
Alveolar ridge augmentation using bFGF-incorporated gelatin sponge and collagen membrane. (a) Preoperative intraoral photograph shows narrow alveolar ridge (arrows); (b) frontal plane of the preoperative dental CT is shown, and (c) bFGF-incorporated gelatin sponge (arrows) is implanted. Inserted photograph demonstrates bFGF-incorporated gelatin sponge, and (d) the narrow alveolar ridge is reconstructed (arrows) 8 months postoperatively.

Also, author et al. showed that bFGF incorporating gelatin sponge (IP: 5.0) promoted a new bone formation in the alveolar ridge defects of dogs [32], and nowadays they have a successful clinical application, although more trials are needed to reach concrete conclusions (Figures 3 and 4). Moreover, author et al. pay special attention to the micrometer-sized protein crystals called polyhedra as DDS matrix of growth factors. Bombyx mori cypovirus is a major pathogen, which causes significant losses in silkworm cocoon harvests because the virus particles are embedded in polyhedra and can remain infectious in harsh environmental conditions for years [134, 135]. But the remarkable stability of polyhedra can be applied on slow-release carriers of cytokines for tissue engineering. Then, we examined healing in critical-sized bone defects by bone morphogenetic protein-2 (BMP-2) encapsulated polyhedra. ACS impregnated with BMP-2 polyhedra had enough osteogenic activity to promote complete healing in critical-sized bone defects, but ACS with a high dose of rhBMP-2 showed incomplete bone healing, indicating that polyhedral microcrystals containing BMP-2 promise to advance the state of the art of bone healing [136].

#### 2.3.2. Synthetic Polymers

Synthetic polymers for carrier (scaffold) of growth factors and aliphatic polyesters such as PLA, PGA, and PCL are most commonly investigated. Growth factors can be covalently bound to polymers or physically entrapped inside a polymer matrix [103]. In either case, they are released into healing sites as the polymer degrades in the physiological environment [34, 100]. They demonstrated the ability to promote bone formation compared with control scaffolds [137–139]. However, there are differences in the bioresorption process of these polymers. The approximate time for the bioresorption of PLA is a few years and that of PGA is 4–6 months, and degradation products of these polymers produce local acid environments [16, 140]. On the other hand, PCL shows relatively slow degradation rate similar to that PLA, but PCL degradation products are easily resorbed through metabolic pathways and do not produce local acid environments that are produced in carriers of PLA or PGA, which may affect the stability of growth factors [141]. Also, PLGAs are often utilized, and a variety of growth factors are encapsulated within the polymer [142–144]. Furthermore, Takaoka et al. have challenged a new carrier by co-polymerization of PEG and aliphatic polyesters: poly-d, l-lactic acid-p-dioxanone-polyethylene glycol block copolymer (PLA-DX-PEG) [145–147]. This carrier exhibited promising degradation characteristics for BMPs delivery systems and new bone formation effectively. Also, they reported that PLA-DX-PEG and porous *β*-TCP composite containing BMP-2 is a promising composite with enough osteogenicity to repair large bone defects [148].

#### 2.3.3. Challenge to Delivery of Multiple Bioactive Molecules

In a living body, bone healing and regeneration are progressed via the action of a number of growth factors [103, 149]. Single growth factor delivery has a number of limitations. Significant efforts have been made in the recent years to develop schemes for combinatorial or sequential delivery of multiple growth factors [150–153]. For example, different-biodegraded particles containing different factors can be embedded and combined into bulk scaffolds, enabling sequential and tighter control of release profiles [152]. Also, titanium wire coated with multiple layers of poly(D,L-lactide) (PDLLA) allowed sequential release of gentamicin (antibiotic), BMP-2, and IGF-I, and this increased bone formation [153]. Furthermore, gelatin or alginate hydrogels are being explored for their utility in sequential release [150, 154, 155]. Highly functional scaffolds incorporating multiple growth factors and controlling their spatial and temporal release will further the progress of craniomaxillofacial bone tissue engineering, but they are still in their infant stage and have not reached even preclinical research.

## 3. Scaffolds for Cellular Bone Tissue Engineering (Bone Tissue Engineering Using Cells)

When the bone defect is larger, bone healing is insufficient or compromised, making it necessary to provide cells from an external source. There are two approaches in which cells are used for bone regeneration. The first approach is to directly transplant osteoprogenitor cells or mesenchymal stem cells (MSCs) together with a scaffold (an external scaffold) to the bone-defected site (*in situ* regeneration) [156–158]. The second approach is to transplant MSCs which are proliferated within three-dimensional scaffolds *in vitro* to the bone defect (extracorporeal tissue engineering or tissue engineering in a narrow sense) [159–161].

### 3.1. Scaffolds for Autogenous Particulate Cancellous Bone and Marrow (PCBM) Transplantation

Particulate cancellous bone and marrow (PCBM) is most commonly used as the source of osteoprogenitor cells or MSCs. Namely, PCBM is rich in osteogenic progenitor cells and bone matrices, and it also has full bone formation ability. In addition, the spontaneous regeneration of donor sites is possible. However, because PCBM does not, by itself, feature structural strength and the ability to hold its shape, it is necessary to provide some kind of framework (external scaffold), which will lead bone formation to the desired shape and which will be able to support the newly formed bone in acquiring to withstand external force. Furthermore, it is desirable for this framework to allow vascular invasion, to biodegrade after the process of bone repair is complete, and to disappear from the living body tissue.

**Figure 5:**
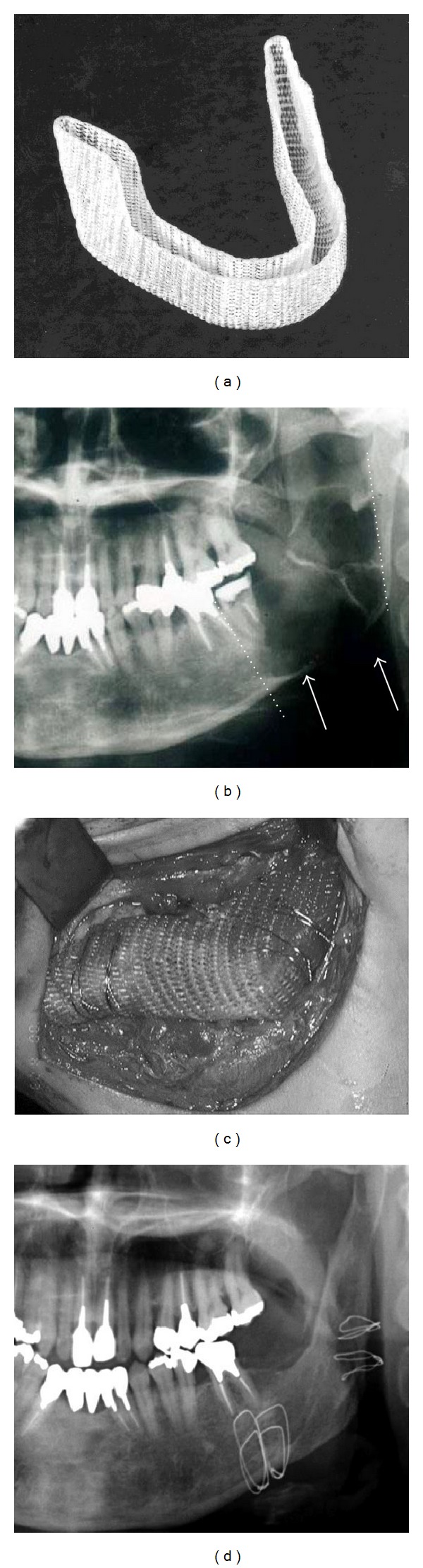
Regeneration of the jaw using PLLA mesh and PCBM [163]. (a) PLLA mesh tray and (b) keratocystic odontogenic tumor of the left mandible, preoperative panoramic X-ray. Multilocular radiolucent area (arrow) and segmental resection line (dotted line). (c) Reconstruction using the PLLA mesh tray and PCBM and (d) X-ray image 1 year and 586 months after the reconstructive surgery. Formation of the matured regenerated bone and mandibular canal.

**Figure 6:**
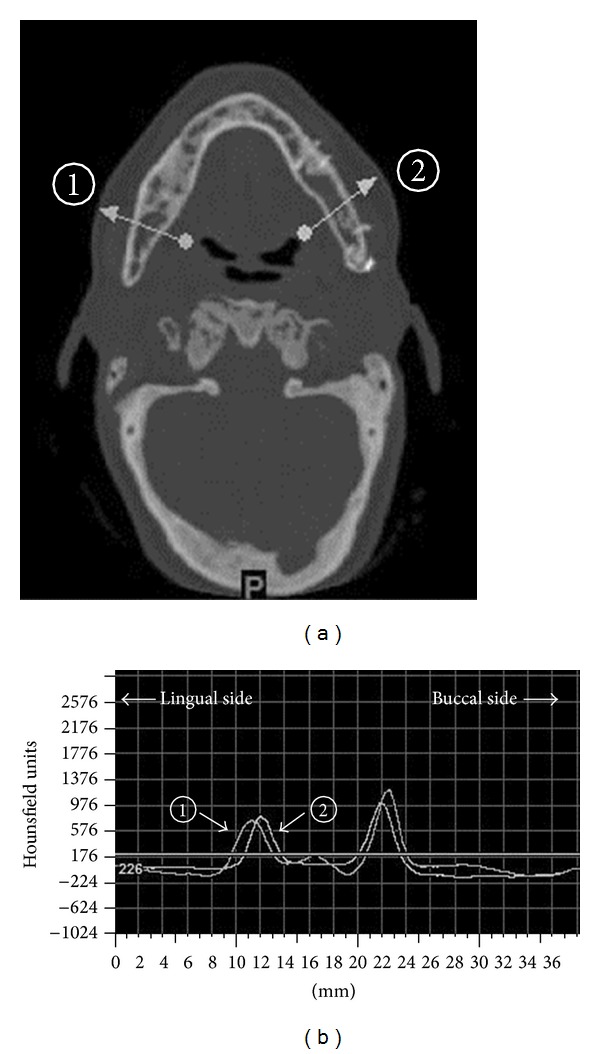
Bone density measurement using CT images and values [163]: (a) the regenerated bone shows no resorption 8 years postoperatively. The bone density was measured in 2 areas (one healthy bone area (1) and one regenerated bone area (2)) (arrows), and (b) the bone density. The peak of the cortical bone density was remarkably not different for the regenerative bone and the healthy bone.

 Author et al. have developed a biodegradable PLLA mesh for the scaffold [162]. The PLLA mesh was made of PLLA monofilaments with diameters of 0.56 and 0.6 mm (Gunze, Ltd., Kyoto, Japan). The PLLA monofilaments were fabricated from PLLA having a molecular weight of 20.5 × 10^4^ Da by spinning at 245°C and drawing at 80°C. These filaments were woven into the mesh. The PLLA mesh was cut with scissors and easily molded by heating it up to approximately 70°C. The author and colleagues reconstructed mandibular defects of 62 patients (22 malignant tumors, 30 benign tumors, 5 cysts, 2 osteomyelitides, 2 traumas, and 1 atrophy of the alveolar ridge) using PLLA mesh and PCBM. The follow-up period was between 9 and 200 months (average 88.2 months) [163]. Consequently, bone regeneration at six months post-operatvely was excellent in 35 cases (57%), good in 17 cases (27%), and poor in 10 cases (16%) (Figures 5 and 6). Bone resorption >20% was observed in only one of 46 cases with a follow-up term of >1 year. There were no signs of or any other adverse effects except in one case where a section of the tray broke off late in the follow-up periods. PLLA is gradually absorbed over 4 to 5 years by nonenzymatic hydrolysis and phagocytosis by macrophages. Bergsma et al. used a PLLA plate to treat a zygomatic fracture and observed nonspecific foreign-body-reactive swelling during PLLA degradation 3 to 5 years after surgery [164]. However, no delayed swelling was observed in our patients. Our PLLA mesh fabricated by weaving PLLA monofilaments achieved a larger contact surface with surrounding tissue than the mere PLLA plate or the perforated porous PLLA plate [165]. This might result in a better balance of fragmentation and absorption. It is concluded that this method is stable and effective due to favorable morphological and functional recovery and low invasiveness. The disadvantages of mandibular reconstruction using a PLLA mesh and PCBM include an increased risk of infection when performing simultaneous reconstruction of soft tissue and bone and its limitations in patients with extensive bilateral defects, poor regional blood circulation, and full-dose irradiation and/or in advanced age with a poor history of bone regeneration. For such cases, the active supply of a regional blood circulation or concomitant use of bioactive factors that induce angiogenesis and promote bone formation is necessary.

### 3.2. Scaffolds (Internal Scaffolds) for *Ex Vivo* Bone Tissue Engineering

Transplanted scaffolds with seeded mesenchymal stem cells (MSCs) (*ex vivo* bone tissue engineering) have been shown to enhance osteogenic capacity and promote bone formation compared with acellular scaffolds in many preclinical trials [166–170]. However, the clinical application is still confined to a small number, not widely. One of the reasons originates in that an adequate three-dimensional scaffold (3D scaffold) for cell seeding has not yet been developed.

The main role of the 3D scaffold is to simulate the extracellular matrix (ECM), which affects cell adhesion, migration, proliferation, and differentiation. Therefore, such a scaffold should permit cell invasion and easy attachment of cells to the scaffold and provide an environment that is suitable for cell proliferation and differentiation. In order to realize this performance, the scaffold should have excellent biocompatibility, sufficient mechanical strength, inclusion of adequately large pore volume, adequate pore interconnectivity with pore sizes large enough to allow continuous tissue growth, and, moreover, the transport properties to allow the influx of nutrients and elimination of waste products, and the scaffold should be biodegradable, which implies that scaffold degrades during the process of tissue, regeneration and it should finally be replaced with fully functional tissue [34, 35, 38, 39]. So far, various 3D scaffolds have been described, but the major materials of such scaffolds used in the craniomaxillofacial field can be classified on the basis of the specific component materials: minerals, natural polymers, synthetic polymers, and composite materials.

#### 3.2.1. Mineral Materials

Among the mineral materials, hydroxyapatite (HAp) is most commonly used because of its excellent biocompatibility, cell attachment capacity, and osteoconductivity, and it is commercially available in the form of porous implants and granular particles with pores [36, 39, 40, 160, 171]. It has been reported that bone marrow stem cells (BMSCs) show proliferation and differentiation into osteoblasts on porous HAp scaffolds in osteogenic medium, and the constructs of HAp and BMSCs form bone *in vivo* [171, 172]. However, implants of porous or large-particle HAp remain in the body for several years [36]; then, the degree of attaining the final purpose of natural bone organization is somewhat limited. There are a few reports of clinical application [161, 173–176].

Instead of porous HAp, porous TCP and BCP have been attracting attention due to their biodegradability [177–180]. However, these porous ceramics are inherently fragile, which may limit their use in load bearing areas such as craniomaxillofacial bones.

#### 3.2.2. Natural Polymer Scaffolds

Natural scaffolds such as collagen type 1, chitosan, calcium alginate, hyaluronic acid, and DBM have been shown to be osteoconductive [181–184]. Among natural polymers, type I collagen that consists of a main component of extracellular matrix of bone is a representative material for the 3D scaffold. It offers excellent cell adhesion and cell affinity, providing a suitable environment for proliferation and differentiation of BMSCs, and bone regeneration was revealed by implantation of type I collagen scaffold with seeded BMSCs into bone defects [185, 186]. However, collagen scaffolds have poor mechanical strength and rapidly dissolve in living tissue. Also, the collagen sponge significantly shrinks during incubation; pore structures are poorly maintained, and they often failed to allow cell ingrowth into inner scaffolds [187]. To overcome these drawbacks, composite scaffolds of collagen and inorganic materials or composite scaffolds of collagen and synthetic polymers have been investigated [187–192].

Addition of HAp to collagen (collagen-HAp composite) could not only improve the stiffness and porous interconnectivity of a collagen scaffold, but it could also enhance its osteogenic potential and promote osteogenesis both *in vitro* and *in vivo* [190, 193, 194]. Niemeyer et al. fabricated a mineralized collagen coated with noncrystal HA, and they cultured human BMSCs on the scaffold [190]. They showed that the seeding efficacy, the expression of the osteogenic marker gene, and the cell infiltration were higher than in *α*-TCP scaffolds. Xu et al. also fabricated a novel biomimetic composite scaffold bioglass-collagen-phosphatidylserine (BG-COL-PS) with a freeze-drying technique [195]. The responses of MSCs to the scaffold exhibited a higher degree of attachment, growth, and osteogenic differentiation than those on BG-COL scaffolds *in vitro*. The *in vivo* results showed that BG-COL-PS composite scaffolds exhibited good biocompatibility and extensive osteoconductivity with the host bone. Moreover, the BG-COL-PS/MSC constructs dramatically enhanced the efficiency of new bone formation than BG-COL/MSC constructs. On the other hand, addition of synthetic polymer to a porous collagen scaffold will provide adequate mechanical strength to the scaffold. Hiraoka et al. developed a sponge that consisted of collagen and PGA fiber (PGA/collagen sponges), which provided reinforcement without impairing biocompatibility [192]. Author et al. fabricated PGA filaments/collagen sponge under the same conditions, and they cultured the sponge seeded with BMSCs for 3 weeks. In contrast to the collagen, the sponge shrunk considerably, and the PGA/collagen sponge maintained most of its original shape during culturing. The PGA/collagen had higher ALP activity and cell number than the collagen sponge. Although the collagen sponge showed cell proliferation only on the surface of the sponge, the PGA/collagen sponge showed it within itself. SEM micrographs showed better attachment onto the PGA/collagen sponge than onto the collagen sponge [187].

#### 3.2.3. Synthetic Polymers

Aliphatic polyesters, including PGA, PLA, PCL, and their copolymers, are the most popular and widely used for 3D scaffolds. The proliferation and differentiation of BMSCs or periosteal cells on scaffolds of nonwoven fabric, mesh, and sponge shape made of these polymers have been investigated both *in vitro* and *in vivo* [196–208]. However, their cell affinity is lower compared with that of collagen, and the osteoconductivity is lower compared with ceramics because of hydrophobic properties. Thus, cellular ingrowth into the scaffold remains insufficient, resulting in poor bone formation in the central part of the sponge-type scaffold. In order to maintain the advantages and to eliminate the disadvantages of synthetic polymers, the challenges to develop functional synthetic polymer-based scaffolds which are combined with natural polymer and ceramics have been undertaken [203, 209–213]. Liu and Ma reported that the compressive modulus of PLLA/HAp composite scaffolds reached the same range as trabecular bone and the new bone formation quite uniformly distributed throughout the composite scaffold in contrast with only surface layer growth in plain polymer scaffolds [213]. Ciapetti et al. described that the dissolution of HAp granules in PCL/HAp composite scaffold released Ca/P ion, influenced cells in the immediate vicinity, induced the redeposition of calcium phosphate, enhanced bone formation, and, moreover, corrected the release of acid from the polymer [203]. Takechi et al., using solvent casting/particulate leaching method, fabricated highly porous 3-dimensional scaffolds (PL-aAC) consisting of biodegradable poly(D,L-lactide-co-glycolic acid) (PLGA) with hydroxyapatite particles containing atelocollagen (aAC) [212]. According to results of examination of its basic properties and biocompatibility both* in vitro *and* in vivo*, PL-aAC scaffolds showed a greater strength and stability than PLGA scaffolds, and superior performance in terms of cell attachment and proliferation as compared with PLGA. They, furthermore, suggested that PL-aAC was more useful for cell transplantation as compared with PLGA for bone tissue engineering.

Also, coating of the surface of synthetic polymer scaffolds with incorporate inorganic material has been investigated. The nature of the material surface determines whether protein molecules can adsorb or not, and how cells attach, and directly affects cellular response, ultimately influencing the rate and quality of new bone formation. Zhang and Ma developed a biomimetic process that allows the *in situ* apatite formation on the internal surface of the pore wall of polymer scaffolds using simulated body fluids (SBFs), which revealed that, in addition to improved osteoconductivity, the mechanical properties of the scaffolds were also significantly improved over the plain polymer scaffolds [214]. SBF-treated HA-added porous PCL scaffold revealed more differentiation of osteoblasts and mineralization than those of plain HA-added microporous PCL *in vitro* [204]. SBF-treated HA-added macroporous PLGA also promoted significantly regeneration of osteoid matrix and mineralized tissue within a rat cranium critical defect compared with a nonmineralized polymer scaffold [215].

#### 3.2.4. Micro- and Nanocomposite Scaffolds Using Nanotechnology

Native ECM of the bone is a hierarchically organized micro- and nanocomposite [35]. It has been shown that bone cells are profoundly influenced by topography of the scaffold [216–219]. To mimic the unique micro- and nanoscale characteristics of natural bone is very important for the design of the higher functional scaffold. The microscale features of natural bone provide a pathway (conduit) for vascularization, nutrients delivery, and cell migration. Therefore, highly porous microscale scaffolds also allow for higher levels of nutrients diffusion, vascularization, and better spatial organization for cell growth and ECM production [220]. In the literatures, a pore size that provides enough nutrient and osteoblast cellular infusion is in the range of 10–400 *μ*m. However, a room of the investigation remains about the optimal porosity and pore size [35, 220–223]. Also, the natural bone ECM has a random configuration. It has been suggested that randomly positioned pores contribute to better cell seeding and better cell aggregation in the scaffolds [221]. However, by the conventional scaffold fabrication (particulate leaching, gas forming, fiber meshes/fiber bonding, phase separation, melt molding, solution casting, and freeze drying), it is impossible to control the pore size, the pore geometry, and the pore distribution in the scaffold [36, 223–227].

The recent development of solid free-form fabrication techniques such as 3D printing and 3D plotter makes it possible to fabricate 3D scaffold with more precise external shape and internal morphology [227]. 

While microscopic porosity plays a key role in the osteoconductivity of a scaffold, the nanoscale structure of the material is considered to primarily influence the osteoinduction and osseointegrativity of the scaffold. Native bone cells interact with nanoscale protein and mineral, and they are predisposed to adhere, grow, proliferate, differentiate, and produce ECMs based on the nanoscale interaction [35, 228]. A variety of nanofabrication techniques have emerged in the recent years. Among them, electrospinning has emerged for fabricating bone-mimetic nanofiber scaffolds. Electro spinning makes it possible to obtain microfibers and nanofibers from polymeric solutions or melts and to fabricate bone-mimetic nanofiber scaffolds [35, 40, 229]. Nanofibers have a large surface area-to-volume ratio and make fabrication of high porous scaffolds possible. These features are favorable for delivery of protein coating or signaling molecules, cell attachment, cell ingrowth, nutrient diffusion, and angiogenesis in the scaffold during the process of bone regeneration [40]. Electrospinning polymeric scaffolds are made with PLA, PGA, PCL, silk fibroin, calcium phosphates, and bioglass, and glass ceramics [40, 230]. In both *in vitro* and *in vivo* studies, it is demonstrated that osteoprogenitor cells differentiate, proliferate, and adhere in synthetic-nanofibrous matrices [230, 231].

#### 3.2.5. Surface Modification of Scaffolds

For more functionality of cell-based scaffold, chemical modification of the scaffold surfaces similar to those of native ECM should be considered as well as modification of geometrical structure of the scaffold. It will enhance cell attachment, differentiation, and proliferation, and it will support ECM synthesis through cell-surface molecular interactions. Researchers are working towards the incorporation of the biologically significant regions of natural proteins into synthetic materials [35, 232, 233]. For example, the surface modifications with the arginin-glycine-aspartic-acid- (RGD-) peptide have been investigated [234–237]. RGD-peptide mediates cell attachment to matrix proteins such as fibronectin, fibrinogen, vitronectin, and osteopontin. Surface modification of synthetic scaffolds using RGD-peptide sequence facilitates the attachment of cells to the scaffold, which leads to enhancement of cell proliferation and differentiation and which results in promoting bone regeneration. Bone sialoprotein is also an RGD-containing protein that is abundant in mineralized tissues at sites of new bone formation. It is considered that bone sialoprotein mediates early bone formation [238]. Chan et al. developed polycaprolactone poly(2-hydroxyethyl methacrylate) (PCL/pHEMA) polymer networks that were surface modified with bone sialoprotein [233]. Osteoblast cell attachment and spreading were enhanced on bone sialoprotein-modified surface compared with that of control surface (unmodified or albuminwen conjugate) that is likely mediated through cell-surface receptors for RGD sequence. These enhanced cell-surface interactions will also enhance cell proliferation, differentiation, and matrix synthesis and will result in promoting the regeneration bone. Furthermore, collagen mimetic scaffold that was composed of glycine-phenylamine-hydroxyproline-glycine-glutamate-arga (GFOR) was developed, which substantially enhanced osteoblast functionality and osseointegration *in vivo* compared with control scaffold [239]. These improvements in the capacity of the scaffold to promote cell-surface of scaffold interactions will contribute to the development of innovative scaffolds for application of cell-based tissue engineering in craniomaxillofacial bone region. In the recent years, the induced pluripotent stem cells (IPSCs) have been effectively induced from dental pulps and expected to be cell sources for tissue engineering. In order to make use of them for regeneration of craniomaxillofacial bone, development of more functional scaffolds is required.

## 4. Summary and Future Direction

To overcome limitations with utilizing autogenous bone grafts as the gold standard treatment for critical-sized defects in craniomaxillofacial bone, many researchers have been challenging bone tissue engineering past two decades. The scaffold is a fundamental element in bone tissue engineering, which plays a role of GBR membrane, temporary bone substitute, DDS of growth factors, or 3D scaffold for cells seeding, cell proliferation, and cell differentiation according to the circumstances of the bone defects. In many cases, it is a principle that the scaffolds should be biodegradable replacements for bone. Although some of them are applied to clinical cases, scaffolds to be clinically completely satisfied have not being developed. Development of more functional scaffold is needed so that it may be applied widely.

Remaining challenges are as follows: (1) development of a scaffold which has adequate mechanical properties throughout bone regeneration, (2) development of a scaffold for DDS which encapsulates growth factors and has closely controlled temporal spatial long-term release profiles with efficacy and nontoxicity, (3) development of a 3D scaffold with the structure mimicking ECM of natural bone, and (4) development of a 3D scaffold which promotes vascularization. In particular, clinical application of cell-based tissue engineering in craniomaxillofacial bone is still very little. More development of high functional composite scaffolds with architecturally elaborate structure mimicking natural ECM is being awaited as well as the establishment of the cell cultivation technology. Fabrication of scaffolds based on recent advances in nanotechnology will make it possible to realize this performance.
